# Preclinical Activity of Datopotamab Deruxtecan (Dato-DXd), an Antibody–Drug Conjugate Targeting TROP2, in Poorly Differentiated Endometrial Carcinomas

**DOI:** 10.1158/2767-9764.CRC-25-0251

**Published:** 2025-09-11

**Authors:** Niccolò G. Santin, Namrata Sethi, Stefania Bellone, Cem Demirkiran, Victoria M. Ettorre, Michelle Greenman, Blair McNamara, Natalia Buza, Tobias M.P. Hartwich, Luca Palmieri, Domenica Lorusso, Alessandro D. Santin

**Affiliations:** 1 Department of Obstetrics, Gynecology and Reproductive Sciences, Yale University School of Medicine, New Haven, Connecticut.; 2 Humanitas Hospital San Pio X, Humanitas University, Milan, Italy.; 3 Department of Pathology, Yale University School of Medicine, New Haven, Connecticut.; 4 Gynecologic Oncology Unit, Department of Woman and Child Health and Public Health, Fondazione Policlinico Universitario A. Gemelli IRCCS, Università Cattolica del Sacro Cuore, Rome, Italy.

## Abstract

Datopotamab deruxtecan (Dato-DXd) is a novel antibody–drug conjugate (ADC) targeting trophoblast antigen-2 (TROP2), a cell surface glycoprotein highly expressed in many epithelial tumors, to deliver DXd, a potent topoisomerase I inhibitor. We evaluated TROP2 expression in primary endometrial cancer cell lines and the activity of Dato-DXd against endometrial cancer cell lines with different TROP2 expression *in vitro* and *in vivo*. TROP2 expression was assessed in nine primary tumor cell lines by flow cytometry. Cell viability after exposure to Dato-DXd was evaluated using flow cytometry–based assays to calculate the IC_50_. Bystander effect assay assessed the viability of TROP2-negative cells when cocultured with high TROP2-expressing cells. Fluorescent anti–phosphorylated histone H2AX antibody was used to demonstrate double-strand DNA breaks. Antibody-dependent cell cytotoxicity was tested *in vitro* using 4-hour chromium release assays. *In vivo* activity of Dato-DXd was evaluated against TROP2-positive endometrial cancer xenografts. A total of 78% (seven of nine) of the primary endometrial cancer cell lines expressed TROP2. Endometrial cancer cell lines expressing TROP2 were significantly more sensitive to Dato-DXd compared with control ADC. Dato-DXd–exposed, TROP2-positive endometrial cancer demonstrated increased double-strand DNA breaks compared with non-binding conjugate exposure. Dato-DXd mediated antibody-dependent cell cytotoxicity against TROP2-positive cell lines and induced significant bystander killing of TROP2-negative tumors when admixed with TROP2-positive tumors. *In vivo*, injection of Dato-DXd was well tolerated and demonstrated impressive tumor growth inhibition against chemotherapy-resistant poorly differentiated endometrial cancer xenografts (*P* < 0.0001). In conclusion, Dato-DXd is a novel ADC with remarkable preclinical activity against poorly differentiated endometrial cancer cell lines overexpressing TROP2. Clinical trials with Dato-DXd in patients with recurrent endometrial cancer are warranted.

**Significance:**

Targeted treatment of aggressive forms of endometrial cancer using the biomarker TROP2 is a significant opportunity for the development of treatments when patients are resistant to other lines of treatment. Here, we present data showing preclinical evidence of effectiveness of this biomarker-targeted therapy in endometrial cancer.

## Introduction

Endometrial cancer is the sixth most common cancer in women worldwide, with 420,368 women diagnosed and 97,723 deaths reported in 2022 (1). Endometrial cancer is the most prevalent gynecologic malignancy in the United States, with 65,950 newly diagnosed cases and 12,650 deaths in 2022 (2, 3). Endometrial cancer incidence and mortality are increasing worldwide, with 69,120 new cancer cases and 13,860 deaths expected in 2025 in the United States alone (2, 3). If current trends continue, the incidence of endometrial cancer will double by 2030 (4). Surgical resection/staging (i.e., hysterectomy with bilateral salpingo-oophorectomy and sentinel lymph node sampling) using an open or minimally invasive (i.e., laparoscopic and/or robotic) approach, followed by radiation, cytotoxic chemotherapy, endocrine, hormonal and/or immunotherapy treatment depending on patients’ recurrence risk, has been the staple of treatment for the last decade.

A histologic classification (i.e., type I estrogen-dependent vs. type II estrogen-independent endometrial cancer; ref. 5) has been used for decades to counsel patients with uterine cancer about prognosis and to guide decision-making with regard to the necessity for adjuvant treatment. Recently, however, genome-wide analyses have revealed the landscape of the complex genomic alterations present in endometrial carcinomas and have provided deeper and more valuable insights into the pathogenesis of this disease. Accordingly, the Bokhman classification has been now superseded by a molecular one classifying endometrial cancer into four groups based on genomic abnormalities: (i) POLE mut: DNA polymerase-ε (POLE) mutation, in which patients harbor ultra-mutated endometrial cancer and have in general a good prognosis, (ii) MMRd: mismatch repair–deficient, in which patients developed a hyper-mutated microsatellite-unstable endometrial cancer and have an intermediate prognosis, (iii) p53abn: protein 53 abnormal expression, which has a poor prognosis, and (iv) NSMP: no specific molecular profile, which has a stage-dependent prognosis (6).

Most cases of endometrial cancer are diagnosed at an early stage and can be treated successfully with surgery alone (5-year survival rate for patients with stage I disease is 90%; refs. 7, 8). However, up to 35% of patients with endometrial cancer are diagnosed with a more aggressive histopathology, such as poorly differentiated endometrioid, copy number–high serous-like carcinoma, clear cell carcinoma, and carcinosarcoma (5, 9). These patients harboring biologically aggressive types of endometrial cancer account for the majority who present with stage III or IV disease and regardless of the use of gold standard chemotherapy treatment (i.e., carboplatin plus paclitaxel) and more recently immune checkpoint inhibitors (ICI) remain uncurable (i.e., 5-year survival rate for stage IVA and IVB endometrial cancer is only 17% and 15%, respectively; refs. 8–11). The identification of novel treatment modalities for patients diagnosed with biologically aggressive endometrial cancer unresponsive or with acquired resistance to chemotherapy and ICI treatments remains an unmet medical need.

Antibody–drug conjugates (ADC) are highly targeted drugs that combine the use of monoclonal antibodies specific to a surface antigen expressed on tumor cells with highly potent anticancer agents linked via a chemical linker. ADCs may therefore optimize tumor targeting *in vivo* while potentially minimizing the side effects of highly toxic chemotherapy agents (12). Many ADCs are currently in late-stage development whereas others are either in early clinical trials or have recently been approved for clinical use by the FDA. For example, trastuzumab deruxtecan (Enhertu; Daiichi Sankyo/AstraZeneca) is currently approved by the FDA and European Medical Agency for the treatment of HER2-positive metastatic solid tumors, while mirvetuximab soravtansine (Elahere; AbbVie Inc.) has been recently approved in patients with recurrent platinum-resistant ovarian cancer.

Human trophoblast cell surface antigen 2 (TROP2) is a 46 kDa transmembrane glycoprotein encoded by the *TACSTD2* gene on chromosome 1p32, which is differentially expressed in a variety of epithelial tumors (13). TROP2 overexpression has been previously demonstrated to represent an independent marker for poor prognosis in multiple human tumors including endometrial endometrioid adenocarcinomas by promoting increased invasion and metastases (14, 15). Importantly, TROP2 is differentially expressed in tumor cells versus healthy tissue cells, making the targeting of TROP2 an attractive option for cancer immunotherapy.

Datopotamab deruxtecan (Dato-DXd) is a novel ADC combining the humanized datopotamab antibody targeting TROP2 with the toxic payload DXd, a topoisomerase I inhibitor (i.e., an exatecan derivative), through a cleavable linker that allows for its time-dependent release in the tumor tissue (16). The drug-to-antibody ratio (DAR) of Dato-DXd is 4:1 (17). Importantly, the tetrapeptide-based cleavable linker of Dato-DXd is cleaved by lysosomal enzymes differentially expressed in tumor cells, ensuring the ADC has stability while in systemic circulation, thus limiting systemic toxicity (18), in addition to allowing for a bystander effect against nearby TROP2-negative tumor cells within the tumor microenvironment. Multiple phase I/II trials have shown promising antitumor activity in non–small cell lung cancer (19), metastatic triple-negative breast cancer (20), and metastatic hormone receptor (HR)–positive, HER2-negative breast cancer (objective response rate, 29%; ref. 21) in TROPION-PanTumor01. Importantly, encouraging clinical activity of Dato-DXd in patients with recurrent endometrial cancer has recently been presented at the ESMO 2024 international meeting (22).

The objective of this study was to evaluate the expression of TROP2 in primary endometrial cancer cell lines. We then sought to examine the preclinical antitumor activity of Dato-DXd *in vitro* and *in vivo* against multiple primary endometrial cancer cell lines and xenografts. We demonstrate for the first time that Dato-DXd is highly active, both *in vitro* as well as *in vivo*, against biologically aggressive endometrial cancer and well tolerated in animals bearing endometrial cancer xenografts.

## Materials and Methods

### Establishment of endometrial cancer cell lines

Approval for this study was obtained through the Institutional Review Board. All patients were consented via written informed consent before tissue/blood collection per institutional guidelines and the Declaration of Helsinki. Nine primary endometrial cancer cell lines were established from fresh tumor biopsy samples, determined to be free of *Mycoplasma* and with limited *in vitro* passages (i.e., less than 50), validated with whole-exome sequencing, and de-identified as described previously by our group (23–26). Cell sample characteristics (i.e., histology, stage, and grade) and patient demographics (i.e., age and ethnicity) are described in Table 1. Tumors were staged according to the International Federation of Gynecology and Obstetrics staging system.

**Table 1:** Cell lines with their demographics and characteristics for primary endometrioid cancers

Cell line	Patient age	Ethnicity	Histology	FIGO^a^ stage	Grade^a^	TROP2 expression^b^	MFI^b^
END(K)82	58	White	Endometrioid	IIIC	G2	3+	833.64
END(K)228	69	White	Endometrioid	IA	G2	1+	22
END(K)283	70	White	Endometrioid	IIIC	G3	1+	32.93
END(K)23	66	Black	Endometrioid	IIIA	G3	1+	47.2
END(K)153	65	White	Endometrioid	IVA	G2	2+	88.85
END(K)202	60	White	Endometrioid	IIIC	G1	0	15.34
END(K)34	64	White	Endometrioid	IB	G2	0	18.08
END(K)254	65	White	Endometrioid	IIIC	G3	3+	1,441.97
END(K)265	50	White	Endometrioid/Clear cell	IIIA	G3	3+	107.13

Abbreviation: FIGO, International Federation of Gynecology and Obstetrics.

MFI 0 to 20 = flow cytometry score 0/negative (Neg); MFI 21 to 50 = flow cytometry score 1+; MFI 51 to 100 = flow cytometry score 2+; and MFI > 100 = flow cytometry score 3+.

a International Federation of Gynecology and Obstetrics staging and grading.

b Mean fluorescence intensity.

### Determination of TROP2 expression in primary endometrial cancer cell lines

Primary endometrial cancer cell lines were tested by flow cytometry for TROP2 expression after being cultured *in vitro* for up to 50 passages. Endometrial cancer cell lines were incubated with 2.5 μg/mL of unconjugated antibody datopotamab for 30 minutes at 4°C and then stained with a phycoerythrin (PE)-conjugated goat anti–human F(ab1)2 immunoglobulin that was used as a secondary reagent (Invitrogen). The data were acquired using CellQuest software (BD Biosciences; RRID: SCR_014489) with FACSCalibur (RRID: SCR_000401). Mean fluorescence intensity (MFI) was evaluated using CellQuest and GraphPad Prism 9 (GraphPad Software, Inc.; RRID: SCR_002798). Cell lines with MFI greater than 100 were determined to have 3+ expression of TROP2, with MFI of 51 to 100 as 2+ expression, 21 to 50 as 1+ expression, and 20 or less were considered negative for TROP2 expression.

### Drugs

Dato-DXd (DS-1062), non-targeting control ADC (CTL-ADC), and unconjugated monoclonal antibody datopotamab IgG were obtained from Daiichi Sankyo Co., Ltd. through a Material Transfer Agreement. Dato-DXd and CTL-ADC were stored at −80°C until use. The agents were serially diluted to prepare concentrations ranging from 0.1 to 50 μg/mL. For the *in vivo* experiments, the drug was diluted with a vehicle (10 mmol/L-acetate buffer and 5% sorbitol, pH 5.5) to ensure a dose of 10 mg/kg in each retro-orbital injection.

### Flow cytometry–based cytotoxicity

Endometrial cancer cell lines were plated in six-well tissue culture plates at a density of 40,000 to 50,000 cells/well in RPMI-1640 media containing L-Glutamine supplemented with 10% FBS, 1% penicillin/streptomycin, and 1% amphotericin. Cells were incubated at 37°C with 5% CO2 for 24 hours. After overnight incubation, non-suspended cells were washed off with PBS and 2 mL of tissue culture media were added to each culture well. Cells were then treated with Dato-DXd and the control ADC at concentrations of 0.1, 0.5, 1.0, 5.0, and 50 μg/mL. After incubation for 72 hours, well contents were harvested in their entirety, centrifuged, and stained with propidium iodide (2 μL of 500 μg/mL stock solution in PBS). Viable cell percentage was then quantified using flow cytometry as a mean ± SEM relative to untreated cells as 100% viable controls. A minimum of three independent experiments per cell line were performed to determine the IC_50_ of Dato-DXd versus CTL-ADC in cancer cell lines.

### Bystander effect assay

Bystander effect assays were performed by admixing TROP2-negative cells [i.e., END(K)34], tagged with carboxyfluorescein succinimidyl ester dye, with TROP2 3+ cells [i.e., END(K)265] in a 1:1 ratio (i.e., 40,000 cells/well of each cell line). Cells were placed in six-well plates and treated with Dato-DXd or CTL-ADC at a concentration of 5 μg/mL after overnight incubation. After an additional 48 hours of incubation, the cells were harvested, washed with PBS, and stained with propidium iodide (2 μL of 500 μg/mL stock solution in PBS) to identify the percentage of live versus dead cells that were present in each well. Analysis of cell viability after treatment was performed using a flow cytometry–based assay, which allowed us to quantify the carboxyfluorescein succinimidyl ester–tagged viable END(K)34 cells versus the non-tagged END(K)265 cells. Using the cell viability data acquired through propidium iodide staining, we were then able to quantify the percentage of viable cells as a mean ± SEM relative to untreated cells as 100% viable controls. A minimum of three independent experiments were performed.

### Double-strand DNA breakage assay

To detect and compare double-strand DNA (dsDNA) breaks, PE-conjugated mouse anti-H2AX (pS139) antibody (BD Biosciences, cat. #562377, RRID: AB_2737611) was used. As done for flow cytometry–based cytotoxicity, three different cell lines [END(K)254 and END(K)265, which were TROP2 3+, and END(K)34, which was TROP2 negative] were first plated and incubated for 24 hours, followed by incubation with 5 μg/mL of control ADC or Dato-DXd for 72 hours. The cells were then harvested and collected. Resuspension was done in 200 μL of fixative buffer (4% paraformaldehyde in PBS 1×) and incubation was done for 15 minutes at room temperature. The cells were washed once with 2 mL of PBS and were then permeabilized using 200 μL of a solution of PBS with 0.5% saponin and 1% BSA. The PE mouse anti-H2AX antibody was added to the cells, 5 μL per sample, followed by incubation on ice for 30 minutes. The cells were washed twice with 2 mL of a solution of PBS with 0.5% of BSA and then analyzed for phosphorylated H2AX as a representation of dsDNA breaks, using BD FACSCalibur.

### Antibody-dependent cellular cytotoxicity

Peripheral blood mononuclear cells (PBMC) were obtained from healthy donors. Cytotoxic activity of Ficoll-Hypaque–separated PBMCs in combination with datopotamab, rituximab (anti-CD20), Dato-DXd, or ADC isotype control (CTL-ADC) against chromium (^51^Cr)-labeled primary endometrial cancer target cell lines at effector-to-target ratios of 5:1 and 10:1 was measured by standard 4-hour ^51^Cr release assay. Tumor cells were exposed to 2.5 μg/mL each of rituximab (anti-CD20), datopotamab, CTL-ADC or Dato-DXd, and the ^51^Cr released from target cells was measured as evidence of tumor cell lysis. As a positive control condition, 0.1% SDS was used to achieve complete lysis of target cells. Chimeric anti-CD20 mAb rituximab was used as the negative control for datopotamab, Dato-DXd, and CTL-ADC in all bioassays. The percentage cytotoxicity of each drug was calculated by the following formula: % cytotoxicity = 100 × (E − S)/(T − S), in which E is the experimental release, S is the spontaneous release by target cells, and T is the maximum release by target cells lysed with 0.1% SDS. A minimum of three replicates were performed. Results are reported as mean ± SEM.

### *In vivo* testing

The *in vivo* antitumor activity of Dato-DXd, CTL-ADC, and datopotamab was tested in a cell line–derived mouse xenograft model using the TROP2 3+ END(K)265 cell line, a grade 3 endometrial cancer with mixed endometrioid and clear cell histology, which has previously shown to be able to consistently grow as xenografts in SCID mice via Protocol #2023-11205 (27). The cell line was injected into 6- to 8-week-old female SCID mice subcutaneously (Envigo). Each mouse was injected with eight million END(K)265 cells suspended in 200 µL of a 1:1 solution of sterile PBS containing cells and Matrigel (BD Biosciences). When the tumor volume reached 0.25 cm^3^, the mice were randomized into four groups: Dato-DXd (10 mg/kg), CTL-ADC (10 mg/kg), datopotamab (10 mg/kg), and control vehicle, with four animals per study group. *In vivo* dosages were chosen based on data published on the bioavailability of the compound in preclinical mouse models (16). All treatment drugs were given as a single retro-orbital injection on day 0. Mice were observed for overall survival (OS) as the primary outcome measure. Tumor volume was measured twice weekly and determined using the formula (A^2^ × B)/2, in which B represented the largest tumor diameter size and A was the smaller perpendicular tumor diameter. Mice were euthanized if tumor volume reached 1.0 cm^3^ as tumor growth exceeding this size was considered inhumane per our Institutional Animal Care and Use Committee (IACUC) protocol. At the conclusion of the study on day 60, the surviving mice were euthanized. Animal care and euthanasia were carried out according to the rules and regulations set forth by the IACUC, in accordance with NIH Guide for the Care and Use of Laboratory Animals. This study was approved by the IACUC.

### Statistical analysis

Statistical analysis was performed using GraphPad Prism 9. The IC_50_ experiments, dsDNA breakage analysis, the bystander assays, and the antibody-dependent cell cytotoxicity (ADCC) experiments were evaluated by a two-tailed paired Student *t* test. One-way ANOVA was used to evaluate significant differences in tumor volumes at specific time points in the *in vivo* experiments. OS data (OS defined as the time of enrollment to either death or tumor volume of 1.0 cm^3^) were analyzed and plotted using Kaplan–Meier survival curves, which were compared for differences using the log-rank test. A two-sided *P* value < 0.05 was considered significant.

### Data availability statement

The data generated in this study are available upon request from the corresponding author. Anonymized individual patient data may be shared with qualified external researchers based on submitted curriculum vitae and reflecting non conflict of interest.

## Results

### Determination of TROP2 expression in primary endometrial cancer cell lines by flow cytometry

Primary endometrial cancer cell lines were evaluated for surface TROP2 protein expression by flow cytometry. Seven of nine (78%) were found to express TROP2, with four of the nine (44%) cell lines demonstrating moderate to strong (i.e., 2+/3+) TROP2 expression (Table 1). Supplementary Figure S1 shows representative flow cytometric analysis of END(K)254 and END(K)265 cell lines, both showing high TROP2 expression, and END(K)34, a primary endometrial cancer cell line showing no TROP2 expression. Based on these results, we selected END(K)254 and END(K)265 as our representative TROP2-overexpressing cell lines, and END(K)34 as our TROP2 non-expressing cell line for the *in vitro* experiments described below. END(K)265 was selected among the TROP2-overexpressing cell lines for the *in vivo* experiments.

### *In vitro* viability assays with Dato-DXd and CTL-ADC in primary endometrial cancer cell lines

**Figure 1:**
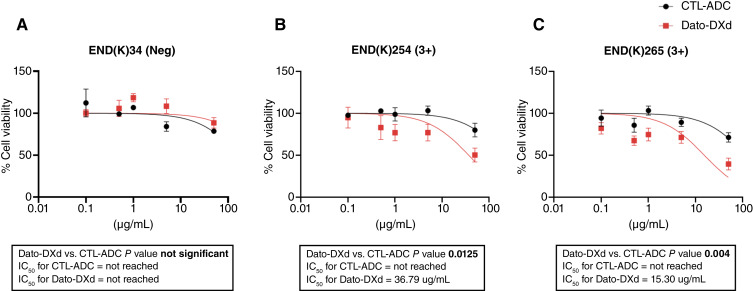
Flow cytometry–based cytotoxicity assay. Determination of Dato-DXd and control ADC IC_50_ (mean SEM) in the tested cell lines. **A,** Cell line with 0 TROP2 expression [i.e., END(K)34] showed no difference in the IC_50_ of Dato-DXd and control ADC. **B** and **C,** Cell lines with high TROP2 expression [3+; i.e., END(K)254 and END(K)265] demonstrated significantly lower IC_50_ for Dato-DXd when compared with control ADC (*P* < 0.05)

We next exposed three primary endometrial cancer cell lines (i.e., two TROP2 positive and one TROP2 negative) to scalar concentrations of Dato-DXd and CTL-ADC with an incubation period of 72 hours. As demonstrated in Fig. 1, Dato-DXd was significantly more potent against TROP2-positive endometrial cancer cell lines when compared with the CTL-ADC (*P* = 0.0125 and *P* = 0.004, respectively; Fig. 1B and C). In contrast, no difference was found in the effect of Dato-DXd versus CTL-ADC on the TROP2-negative END(K)34 endometrial cancer cell line (*P* > 0.05). As demonstrated in Fig. 1A, IC_50_ was not reached using both reagents against the TROP2-negative cell line.

### Bystander effect *in vitro*

**Figure 2:**
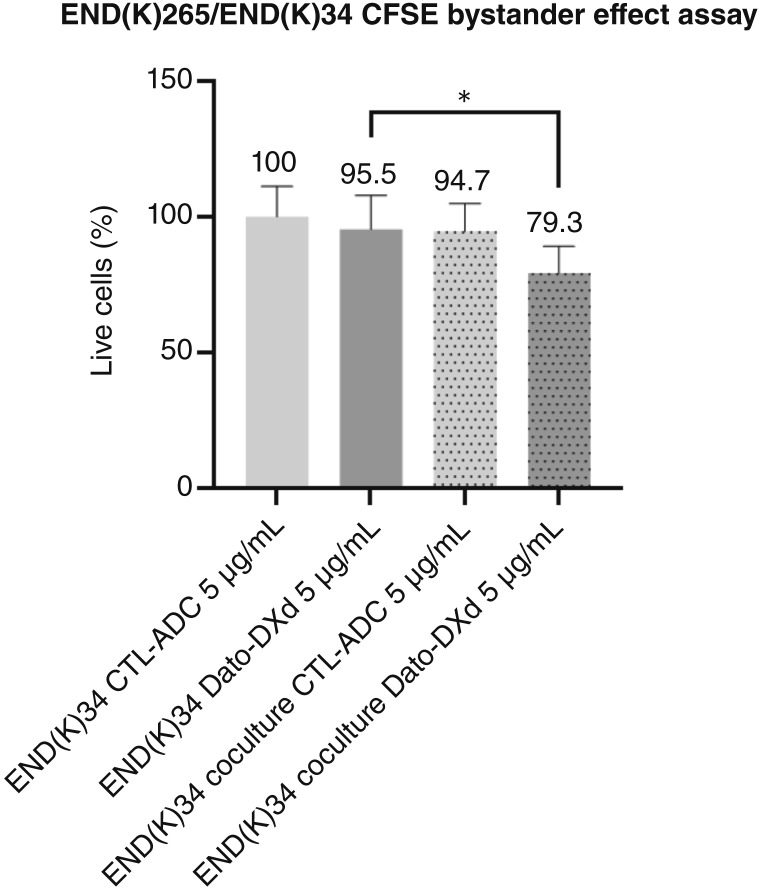
Bystander effect assay. Bystander killing effect was evaluated by admixing END(K)265 (3+ TROP2 expression) *in vitro* with carboxyfluorescein succinimidyl ester (CFSE)–stained END(K)34 cells (0 TROP2 expression). At dose 5 μg/mL, a significant increase in cytotoxicity of END(K)34 cells was seen when END(K)34 and END(K)265 were cultured together and treated with Dato-DXd when compared with ADC control–treated cocultures (*P* = 0.0167). In addition, a significant increase in cytotoxicity of END(K)34 cells was seen when the cocultures were treated with Dato-DXd as compared with isolated END(K)34 cells treated with Dato-DXd (*P* = 0.0329; *, *P* < 0.05)

To evaluate the ability of Dato-DXd to induce a bystander killing effect against endometrial cancer with heterogeneous TROP2 expression, we tested the ADC activity by admixing END(K)265 cells (i.e., 3+ TROP2 expression) with negative TROP2-expressing cells [i.e., END(K)34] for 48 hours. As representatively described in Fig. 2 and Supplementary Fig. S2, a significant increase in cytotoxicity against END(K)34 cells was seen when END(K)34 and END(K)265 were cultured together and treated with 5 μg/mL of Dato-DXd when compared with 5 μg/mL CTL-ADC–treated cocultures (Fig. 2, *P* = 0.0167). Additionally, when tumor cell killing of END(K)34 treated with Dato-DXd was compared with the killing of END(K)34 cells cocultured with END(K)265 and treated with Dato-DXd at the same dose, a significantly higher cell killing activity was detected in the coculture (*P* = 0.0329). Instead, minimal, nonsignificant bystander killing was detected against TROP2 non-expressing END(K)34 cells when the cocultures were treated with CTL-ADC (i.e., 94.7% live cells; Fig. 2).

### *In vitro* dsDNA break assay after treatment with Dato-DXd

**Figure 3:**
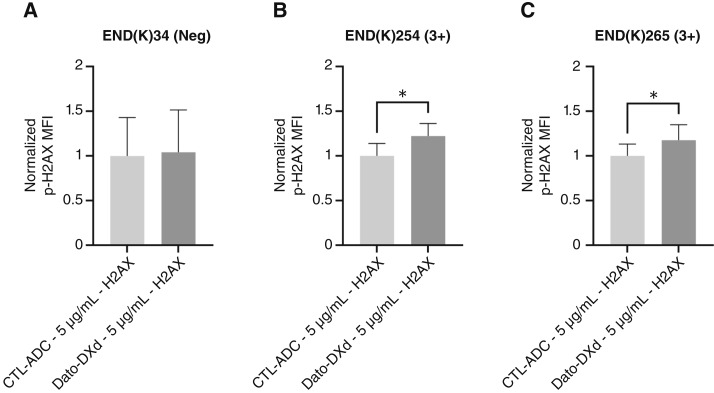
Phosphorylation of histone H2AX as a marker of dsDNA breaks induced from cell internalization of Dato-DΧd. **A,** TROP2-negative cell line END(K)34 does not show statistically significant difference in p-H2AX MFI when treated with 5 μg/mL Dato-DΧd vs. when treated with 5 μg/mL control ADC (*P* > 0.05). **B** and **C,** Increase in p-H2AX MFI is statistically significant when high (3+) TROP2-expressing cell lines END(K)254 and END(K)265 are treated with 5 μg/mL Dato-DΧd vs. 5 μg/mL control ADC (*P* = 0.0156 and *P* = 0.0427; *, *P* < 0.05). The bar graphs show normalized MFI values. Error bars indicate SEM

Phosphorylation of histone H2AX (i.e., γ-H2AX) was used as a marker indicative of dsDNA breaks. We performed a standardized assay using a PE-conjugated mouse anti-H2AX (pS139) antibody to evaluate/confirm increased phosphorylation of histone H2AX in the TROP2 positive END(K)254 and END(K)265 cell lines treated with Dato-DXd when compared with the CTL-ADC. As depicted in Fig. 3, there was a significant increase in H2AX phosphorylation when END(K)254 cells and END(K)265 cells were incubated with 5 μg/mL of Dato-DXd compared with the same dosage of CTL-ADC (*P* = 0.0156 and *P* = 0.0427, respectively; Fig. 3B and C). In contrast, no statistically significant difference was detected when the TROP2-negative END(K)34 cell line was treated with Dato-DXd compared with CTL-ADC (*P* > 0.05; Fig. 3A).

### Dato-DXd and datopotamab induce strong ADCC against TROP2-positive primary endometrial cancer cell lines

**Figure 4:**
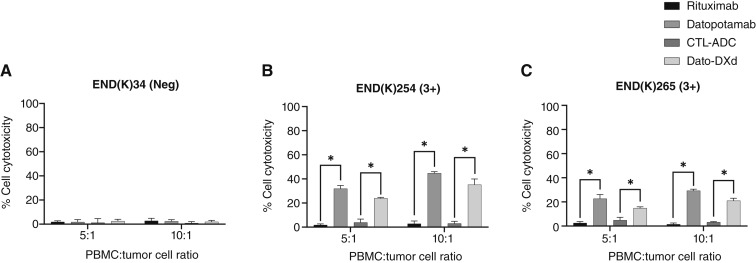
ADCC results of Dato-DXd, CTL-ADC, datopotamab, and rituximab (anti-CD20) in three representative endometrial cancer cell lines [(**A**) END(K)34 TROP2-negative cell line, (**B**) END(K)254 TROP2 3+ cell line, and (**C**) END(K)265 TROP2 3+ cell line] in the presence of PBMCs. Significant ADCC was detectable only against the TROP2-positive cell lines (**B** and **C**; *, *P* < 0.05)

Three representative primary endometrial cancer cell lines (two TROP2 3+ and one TROP2 non-expressing) were tested for their sensitivity to ADCC when challenged with heterologous PBMCs from healthy donors in standard 4-hour ^51^Cr release assays. We found all three endometrial cancer cell lines to be resistant to PBMC-mediated cytotoxicity when incubated in the presence of the isotype control antibody (rituximab; 2.5 μg/mL) at effector-to-target ratios of 5:1 and 10:1 (Fig. 4). We also investigated the sensitivity of endometrial cancer cell lines to heterologous PBMCs in the presence of Dato-DXd, CTL-ADC, and unconjugated datopotamab at 2.5 μg/mL. Datopotamab and Dato-DXd, but not CTL-ADC, were highly effective in inducing ADCC against primary endometrial cancer cell lines expressing TROP2 at high levels [i.e., END(K)254 and END(K)265; Fig. 4B and C]. More specifically, for END(K)254, the *P* value of datopotamab versus rituximab was 0.0041 for ratio 5:1 and 0.0004 for ratio 10:1, whereas for Dato-DXd versus CTL-ADC, the *P* value was 0.0052 for ratio 5:1 and 0.0027 for ratio 10:1. For END(K)265, the *P* value of datopotamab versus rituximab was 0.046 for ratio 5:1 and 0.0022 for ratio 10:1, whereas for Dato-DXd versus CTL-ADC, *P* value was 0.0387 for ratio 5:1 and 0.0056 for ratio 10:1. In contrast, neither the unconjugated antibodies nor the ADCs induced significant ADCC against the TROP2-negative cell line END(K)34 (all *P* values > 0.05; Fig. 4A).

### Antitumor activity of Dato-DXd in a TROP2-positive endometrial cancer xenograft model

**Figure 5:**
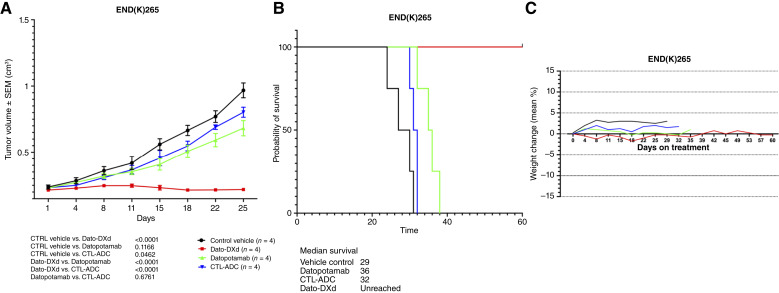
Antitumor activity in mice inoculated with TROP2 3+ xenograft tumor models. **A,** PDX TROP2 3+ END(K)265 after one single retro-orbital injection with Dato-DXd compared with controls, including CTL-ADC, datopotamab, and control vehicle. A significant difference in tumor growth inhibition was detected beginning on day 8 (*P* < 0.05) in Dato-DXd–treated group when compared with the other control groups. **B,** OS of Dato-DXd was compared with the one of datopotamab, CTL-ADC, and control vehicle. The median survival for the Dato-DXd group was unreached, as all mice were alive, compared with 36 days for datopotamab, 32 days for CTL-ADC, and 29 days for control vehicle. **C,** Mouse weight change during the whole duration of treatment

Because of the high TROP2 expression as well as the consistent engraftment of the primary END(K)265 tumor cell lines in SCID mice (27), the *in vivo* experiments comparing the antitumor activity of Dato-DXd, CTL-ADC, datopotamab, and control vehicle were performed using END(K)265 xenograft models (i.e., mixed endometrioid adenocarcinoma/clear cell). As described previously, animals were monitored and randomized into four treatment groups after implanted tumor cells grew to a volume of 0.25 cm^3^, with four animals per treatment group. Treatment with a single retro-orbital injection of Dato-DXd (day 0) showed significant inhibition of tumor growth in these mice (Fig. 5). By day 8, PDX mice treated with Dato-DXd had significantly decreased/stable growth when compared with all three other treatment groups, which experienced continuous linear tumor growth from day 0. The Dato-DXd–mediated inhibition of tumor growth was evident at all subsequent time-points up to day 32 when the CTL-ADC animals were euthanized because of disease progression (i.e., tumor volume >1.0 cm^3^). The overall uncorrected Fisher LSD test demonstrated significant tumor growth reduction in Dato-DXd–treated mice compared with datopotamab (*P* < 0.0001), CTL-ADC (*P* < 0.0001), and control vehicle (*P* < 0.0001) when analyzed at day 32 (Fig. 5A). For END(K)265 TROP2 3+ xenografted mice treated with Dato-DXd, the median OS was significantly improved compared with the other cohorts. The median survival for Dato-DXd was not reached at day 60 as all mice were alive. In contrast, median survival for datopotamab-treated PDX was 36 days, control vehicle 29 days, and CTL-ADC 32 days, respectively. The difference in OS curves between Dato-DXd and three controls was statistically significant (i.e., *P* < 0.0001 when comparing Dato-DXd with each control group individually; Fig. 5B). Importantly, as demonstrated in Fig. 5C, the treatment was well tolerated with no significant differences in weight variations between the four different groups.

## Discussion

Effective treatment options for patients with recurrent, metastatic, chemotherapy-resistant endometrial cancer remain limited. Consequently, developing novel treatment strategies for these patients, while minimizing toxic side effects, represents an unmet need and opportunity in the field of gynecologic oncology. TROP2 is highly expressed in a variety of human epithelial tumors, including cervical, uterine, and ovarian cancers, conferring tumor cells with increased proliferation and cell migration properties (14, 28–31). Importantly, because of its differential overexpression in human tumors when compared with normal cells, TROP2 may represent an excellent target for selective therapeutics such as ADCs. Previous work in our lab has reported TROP2 expression in other biologically aggressive uterine cancers, such as uterine serous carcinoma (USC; ref. 32). Although poorly differentiated endometrial endometrioid carcinoma, similarly to USC, may often be resistant/refractory to standard treatment modalities, these tumors are molecularly different from USC (6) and, accordingly, warrant separate analysis for TROP2 expression.

Accordingly, in this study, we evaluated TROP2 expression level in multiple primary endometrial cancer cell lines by flow cytometry. Importantly, we demonstrated high *in vitro* sensitivity to Dato-DXd exposure in TROP2-positive endometrial cancer cell lines when compared with CTL-ADC. In contrast, no differences were found in the TROP2-negative tumor cell line. These results demonstrate that TROP2 receptor expression is required on tumor cells for the induction of Dato-DXd cytotoxic activity against endometrial cancer.

Next, we demonstrated that tumor cell killing by Dato-DXd may not only be limited to the internalization of the ADC and the consequent intracellular release of the toxic payload DXd but is also potentially mediated by immune system cells, such as NK cells expressing CD16/FcγRIIIA (i.e., Fc co-receptor). Consistent with this view, both Dato-DXd and datopotamab demonstrated significant ADCC against TROP2-positive endometrial cancer cell lines in the presence of PBMCs whereas non-targeting CTL-ADC generated only low cytotoxicity. The killing activity was TROP2 specific as demonstrated by the negligible ADCC induced by Dato-DXd against the TROP2-negative tumor. These findings are in agreement with the results recently published by our group evaluating the activity of the humanized anti-TROP2 monoclonal antibody datopotamab and Dato-DXd in ovarian cancer (33).

Endometrial cancer is a histologically heterogeneous disease and, accordingly, the expression of TROP2, similarly to other surface markers, may not be uniformly expressed on endometrial cancer cells. Importantly, because of the cleavable linker, the DXd payload of the ADC may be released both intracellularly as well as in the tumor microenvironment, thereby allowing for the delivery of therapeutic concentrations of the drug in bystander cells not directly targeted by the ADC (33). Consistent with this view, TROP2-positive tumor cells may be killed by the intracellular uptake of DXd whereas adjacent cells (i.e., TROP2-negative tumor and/or endothelial/stromal cells) may be damaged by its extracellular release. To validate this hypothesis, we performed *in vitro* experiments admixing TROP2-positive with TROP2-negative tumor cells before exposing them to Dato-DXd. We consistently found Dato-DXd to induce a significant bystander killing against TROP2-negative tumor cells only when admixed with TROP2-overexpressing tumor cells, indicating that the presence of TROP2 receptors on the admixed tumor cell populations was essential to this effect. Importantly, in all experiments with TROP2-negative tumor cells admixed with TROP2-overexpressing cells, Dato-DXd induced significantly higher killing when compared with the CTL-ADC, confirming the specificity of the ADC against TROP2-positive targets. These results strongly suggest that Dato-DXd may also be active in the treatment of patients with advanced/recurrent endometrial cancer harboring tumors with heterogeneous TROP2 expression.

dsDNA breaks were assessed using PE mouse anti-H2AX antibody by checking for increased histone phosphorylation of END(K)254 and END(K)265 (i.e., TROP2 overexpressing) cells after treatment with Dato-DXd when compared with CTL-ADC. This flow cytometry assay provided evidence that the cytotoxicity caused by Dato-DXd is likely triggered by the internalization of the ADC, followed by the release of the toxic payload causing both DNA fragmentation and induction of apoptosis in TROP2-expressing endometrial cancer cells.

Importantly, in *in vivo* experiments with TROP2-positive endometrial cancer xenografts established from a patient with a biologically aggressive tumor (i.e., mixed clear cells and G3 endometrioid histology), we were able to demonstrate that injection of Dato-DXd is highly effective in inducing regression of endometrial cancer xenografts. Indeed, a single administration of Dato-DXd (10 mg/kg) caused a statistically significant difference in END(K)265 tumor growth inhibition when compared with control ADC. In this regard, a possible limitation in the use of Dato-DXd in the clinical setting is its potential toxicity. It is therefore worth noting that no evidence of *in vivo* acute or chronic toxicity was detected in animals treated with Dato-DXd for the entire duration of the study. These results demonstrate for the first time *in vivo* activity of Dato-DXd against biologically aggressive and poorly differentiated endometrial cancer and strongly support the use of this ADC in clinical trials.

Taken together, our preclinical *in vitro* and *in vivo* results recently reported using ovarian cancer models (33) and in this article with biologically aggressive endometrial cancer, combined with the results from multiple phase I/II clinical studies, we demonstrated acceptable toxicity and encouraging therapeutic activity of Dato-DXd against multiple recurrent epithelial tumors, including but not limited to non–small cell lung cancer and metastatic triple-negative breast cancer. On January 17, 2025, the FDA approved the use of Dato-DXd for adult patients with unresectable or metastatic, HR-positive, HER2-negative (IHC 0, IHC 1+, or IHC 2+/ISH−) breast cancer who have received prior endocrine-based therapy and chemotherapy for unresectable or metastatic disease based on the efficacy and safety results obtained from the TROPION-Breast01 (NCT05104866) trial (34).

Until the recent Dato-DXd approval, the only FDA-approved TROP2-directed ADC was sacituzumab govitecan (35, 36). Indeed, sacituzumab govitecan is approved for the treatment of metastatic HR-positive HER2-negative breast cancer and metastatic triple-negative breast cancer (37, 38). Although clinical outcomes with sacituzumab govitecan have been promising in patients with endometrial cancer (39), Dato-DXd may offer an even more effective and potentially less toxic alternative as a TROP2-targeted ADC. One key difference is that the toxic payload of Dato-DXd, an exatecan derivative, is over 10 times more potent than the irinotecan metabolite SN-38, which is the toxic payload of sacituzumab govitecan (40). Moreover, Dato-DXd has a significantly longer half-life in human circulation (45.1 ± 13.9 hours) compared with sacituzumab govitecan’s 11 to 15 hours. This extended half-life allows for a more sustained exposure of tumoral tissues to the ADC, enabling administration every 21 days. In contrast, sacituzumab govitecan requires dosing on both days 1 and 8 of a 21-day cycle, increasing the risk of side effects (16). Finally, to improve its therapeutic window and reduce systemic toxicity, Dato-DXd was designed with a lower DAR of 4:1 compared with sacituzumab govitecan’s 7.6:1. This adjustment was made because a higher DAR (i.e., 7:1) showed less favorable safety profiles in preclinical studies with cynomolgus monkeys (16).

### Conclusions

Our previous research work and current results demonstrate that TROP2 is overexpressed in a large percentage of endometrial cancer cases and that primary endometrial cancer cell lines overexpressing TROP2 are highly susceptible to killing *in vitro* by Dato-DXd. This ADC may induce significant ADCC against TROP2-positive endometrial cancer cells in the presence of effector cells (i.e., NK cells), and additionally, Dato-DXd allows for a significant bystander killing effect, which may aid in the treatment of tumors with heterogeneous antigen expression. Finally, endometrial cancer xenografts overexpressing TROP2 are highly sensitive to Dato-DXd *in vivo*. Taken together, these preclinical results, combined with recent phase I/II data demonstrating significant clinical responses in multiple solid tumors resistant to chemotherapy including advanced endometrial cancer, strongly support the design of clinical trials of Dato-DXd in patients with TROP2-expressing biologically aggressive endometrial cancer in progression after chemotherapy and ICIs.

## Supplementary Material

Supplemental Figure 1 Supplemental Figure 1. Representative flow cytometry histograms of the primary EC cell lines used in this experiment.

Supplemental Figure 2 Supplemental Figure 2. Representative flow cytometry image overlay
