# Supplementary figures and images for: Preclinical Activity of Datopotamab Deruxtecan (Dato-DXd), an Antibody–Drug Conjugate Targeting TROP2, in Poorly Differentiated Endometrial Carcinomas
Source: Cancer Res Commun. 2025 Sep 11;5(9):1611–20. doi: 10.1158/2767-9764.CRC-25-0251 (PMC12423748; doi:10.1158/2767-9764.CRC-25-0251)

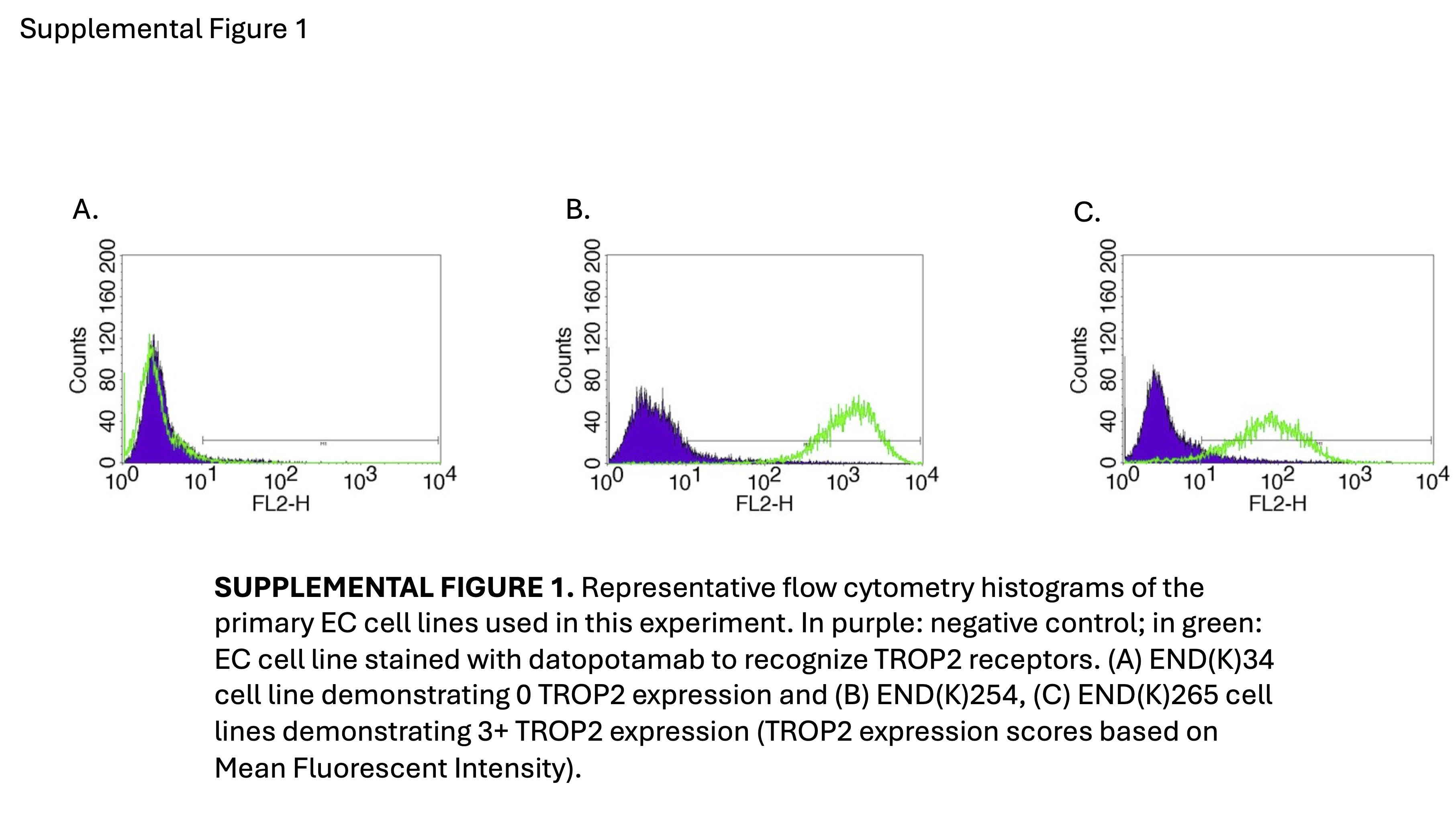

Supplement: Supplemental Figure 1 — Representative flow cytometry histograms of the primary EC cell lines used in this experiment. [file crc-25-0251_supplemental_figure_1_suppsf1.png]

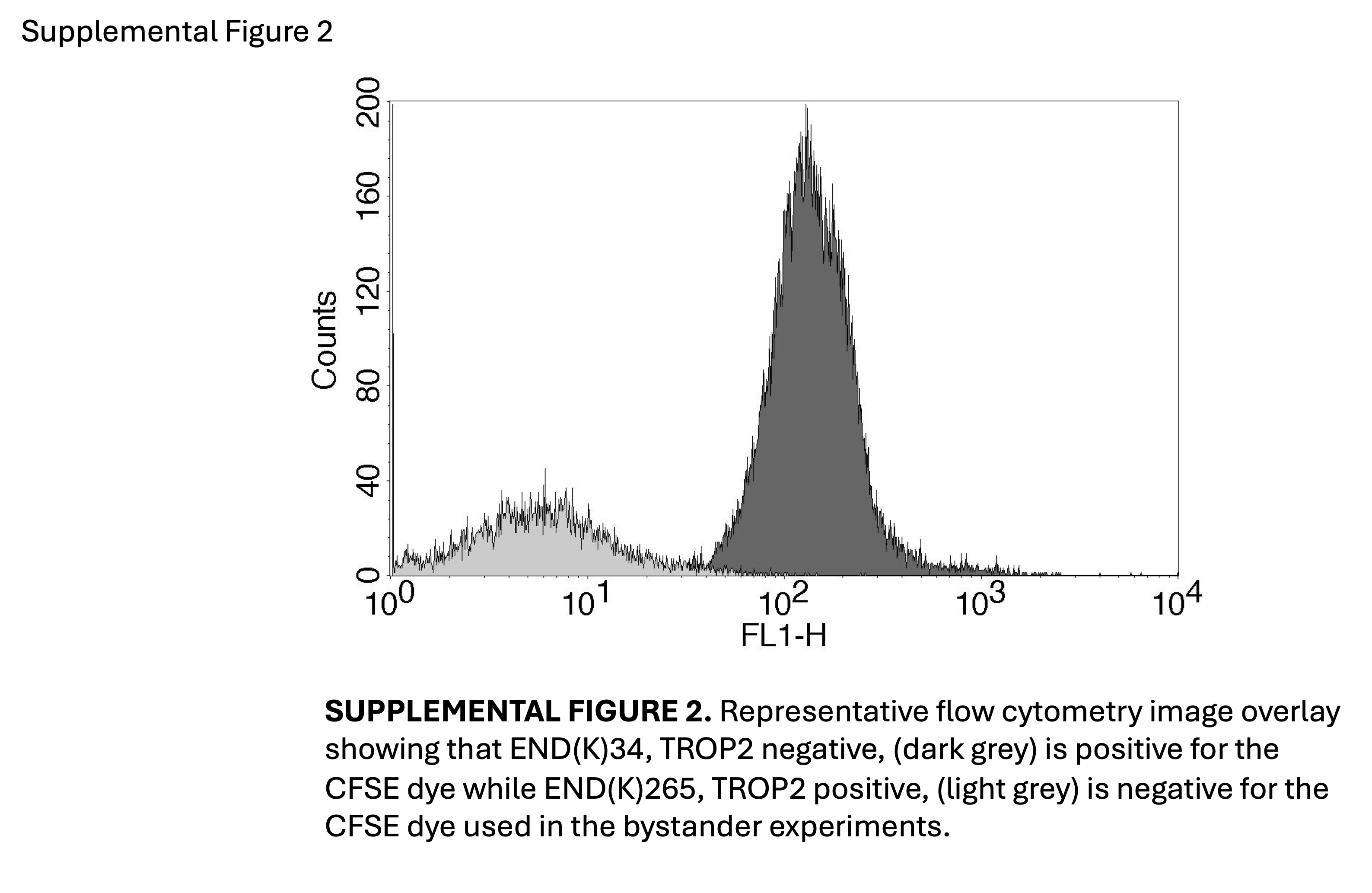

Supplement: Supplemental Figure 2 — Representative flow cytometry image overlay [file crc-25-0251_supplemental_figure_2_suppsf2.png]
